# COVID-19, students satisfaction about e-learning and academic achievement: Mediating analysis of online influencing factors

**DOI:** 10.3389/fpsyg.2022.948061

**Published:** 2022-08-22

**Authors:** Muhammad Younas, Uzma Noor, Xiaoyong Zhou, Rashid Menhas, Xu Qingyu

**Affiliations:** ^1^School of Education, Soochow University, Suzhou, China; ^2^School of Foreign Languages, East China Normal University, Shanghai, China; ^3^Research Center of Sport and Social Sciences, Soochow University, Suzhou, China

**Keywords:** e-learning, students satisfaction, academic achievement, online influencing factors, COVID-19

## Abstract

**Background:**

The current study examines student satisfaction with e-learning, the adaption of online learning channels, digital competency of students' involvement, and academic achievement during COVID-19.

**Purpose:**

The purpose of this study is to examine the online influencing components for learning among University students in Pakistan during the COVID-19 Pandemic.

**Methods:**

The study population comprised Pakistani University students in Punjab province who tooke online lessons throughout the epidemic. In accordance with the study's purpose, a questionnaire survey was employed to gather primary data. SPSS-23 is used for analyzing the demographic data, and cleaning and preparing data for testing hypotheses. SmartPLS 3.0 was used to investigate the suggested study framework using structural equation modeling (SEM).

**Results:**

The analysis of the SEM model shows that all planned hypotheses (Adaptation of Online Education Channels -> Satisfaction about E-learning, COVID-19 Pandemic -> Adaptation of Online Education Channels, COVID-19 Pandemic -> Digital Competence, COVID-19 Pandemic -> Motivation for Online Learning, COVID-19 Pandemic -> Willingness for Online Learning, Digital Competence -> Satisfaction about E-learning, Motivation for Online Learning -> Satisfaction about E-learning, Satisfaction about E-learning -> Academic Achievement, Willingness for Online Learning -> Satisfaction about E-learning) are confirmed.

**Conclusion:**

The results linked e-learning satisfaction to academic success and Pakistani students who utilized e-learning throughout the outbreak reported higher levels of academic satisfaction and achievement.

## Introduction

The World Health Organization (WHO) considered COVID-19 a pandemic in 2020, impacting millions of children and educators globally, and various technological applications have been deployed in the epidemic age to facilitate learning (Nainggolan, 2021; Shahzad et al., 2021). Online learning motivation is a mediating factor in the COVID-19 pandemic condition; when conventional learning/teaching are no longer viable options, online learning may help students continue their education (Adedoyin and Soykan, 2020; Rahman et al., 2021). In a less digitalized economy, mostly higher education institutions changed from face-to-face learning to emergency remote teaching, emphasizing the enabling variables that impact the deployment of e-learning systems during the COVID-19 epidemic (Jan, 2020; Edem Adzovie and Jibril, 2022). The educational integration of technology was tested because of the necessity to establish emergency remote education due to COVID-19 which was implemented to keep students and instructors educated during challenging periods of lockdown (Wang et al., 2020; Valverde-Berrocoso et al., 2021).

### Students' satisfaction with e-learning during COVID-19

Before the COVID-19 epidemic, researchers looked at what made students stay with online learning. As a result of pandemic circumstances, students' participation in academic activities improves before favorably impacting their contentment (Kim and Kim, 2021; Dalipi et al., 2022; Ngah et al., 2022). During the COVID-19 crisis, informal digital learning is critical for students as it investigates the link between digital competency and academic participation in higher education from diverse cultural backgrounds (He and Li, 2019; Heidari et al., 2021). In this unexpected time, the swift migration from conventional face-to-face learning to online learning has been seen as a standard revolution in higher education (Shah et al., 2021). With the COVID-19 epidemic, digital technologies have become an inescapable and vital aspect of learning, and colleges worldwide have suddenly halted face-to-face teaching and resorted to technology-mediated teaching (Okoye et al., 2021; Vladova et al., 2021). Some studies examine the student e-learning satisfaction during the COVID-19 epidemic and the mediation effects of crucial variables, including learning stress and motivation to learn (Mohd Satar et al., 2021; Ong et al., 2021).

### Students' motivation and willingness for e-learning

Universities have started educating students online and are keen to achieve improved learning results utilizing e-interactive learning capabilities (Abou El-Seoud et al., 2014). As part of the COVID-19 study, researchers look at the attitudes of students as well as academic staff toward online learning and distance education and the aspects that influence students' preparedness to continue using an online learning system (Falfushynska et al., 2021; Mabrur et al., 2021; Ngah et al., 2022). Preliminary studies show that e-influence, learning students' interest in utilizing e-learning resources, and their performance and motives impacted their desire to embrace e-learning (Chang et al., 2017; Radha et al., 2020). China has initially employed e-learning strategies by marketing and executing online learning courses in Chinese colleges and universities, and students have willingly accepted this move as new normal (Jin et al., 2021). Previous research analyzes the primary elements that encourage medical students' acceptance and opinions of e-learning during the COVID-19 closing period (Almaiah et al., 2020; Ibrahim et al., 2021). However, despite the problems that come with such a dramatic shift in education, e-learning has shown to be the best solution to the COVID-19 epidemic (Hamdan et al., 2020; Maatuk et al., 2022). The present research investigates the association between student satisfaction with e-learning and academic achievement during COVID-19 in developing nations. Second, the study addresses the adaptation of online learning channels and digital competence of students' appointments in the association between students' satisfaction with e-learning and academic achievements.

## Literature review

In response to the COVID-19 epidemic, China urged millions of full-time students to resume online. Indonesian education examines the necessity for an online model during the COVID-19 epidemic and how educational institutions might employ current resources to bring formal education online (Li and Lalani, 2020; Manca and Meluzzi, 2020; Cahaya et al., 2022). Educational institutions have been compelled to adopt alternate teaching and learning methodologies during the present epidemic, including a complete transfer to online learning. Both professors and students feel that online education is beneficial (Almahasees et al., 2021; Anderton et al., 2021). The continuing COVID-19 epidemic forced the cessation of educational operations, revealing both enabling and hindering elements for successful learning and communication technology integration into online course design and delivery (Khalil et al., 2020; Meletiou-Mavrotheris et al., 2021). Various barriers prevent underprivileged students from entirely using the potential of e-learning, and studies show a statistically significant association between internet gadget availability and utilization (Mpungose, 2020; Segbenya et al., 2022).

During the pandemic of COVID-19, it examined and assessed the impact that e-learning had on the psychological distress of students (Hasan and Bao, 2020). COVID-19 demanded restraint and isolation, affecting teacher–student interactions. Computer-based learning has replaced classroom education and one-on-one engagement. During the continuing COVID-19 epidemic, it is important to analyze University students' perceptions and preparation for online learning (Khan et al., 2021). It is the goal of the investigation to look at how students' online learning experiences and overall satisfaction with their University's brand image are influenced by the use of ICT (Shehzadi et al., 2021; Younas et al., 2022). Several studies examined how college students see themselves as adopting, using, and accepting emergency online learning and the implementation of their online system, and how they make proper decisions to allow more University students to embrace e-learning (Jameel et al., 2020; Patricia Aguilera-Hermida, 2020). Technological advances have affected web technologies and the e-learning process and demand initiatives to leverage the full capabilities of technical innovation to improve e-learning systems and their benefits (Yaw Obeng and Coleman, 2020).

Online teaching helps higher education to reduce cross-contagion in classrooms, and it measures online student satisfaction to assure online teaching quality during the pandemic (Zhang et al., 2021). Online learning is becoming increasingly common during the COVID-19 epidemic period as a means of enhancing students' academic performance in the target language, while student engagement has been found to be an essential factor, and these studies review the literature on online teaching and learning strategies in teacher education (Carrillo and Flores, 2020; Luan et al., 2020). A number of studies have looked at the connections between students' ability to self-regulate, their overall well-being, and their academic performance when taking campus-based courses via distance learning and how teachers can use this cutting-edge technology to get better results from their students by incorporating text, sounds, and visual graphics into their lessons (Muhammad et al., 2020; Yavuzalp and Bahcivan, 2021). To affect students' motivation and aspirations, the usage of e-learning has risen significantly in just a few years and emphasizes the significance of ongoing education for instructors (Paechter et al., 2010).

### Statement of the study

**Figure 1:**
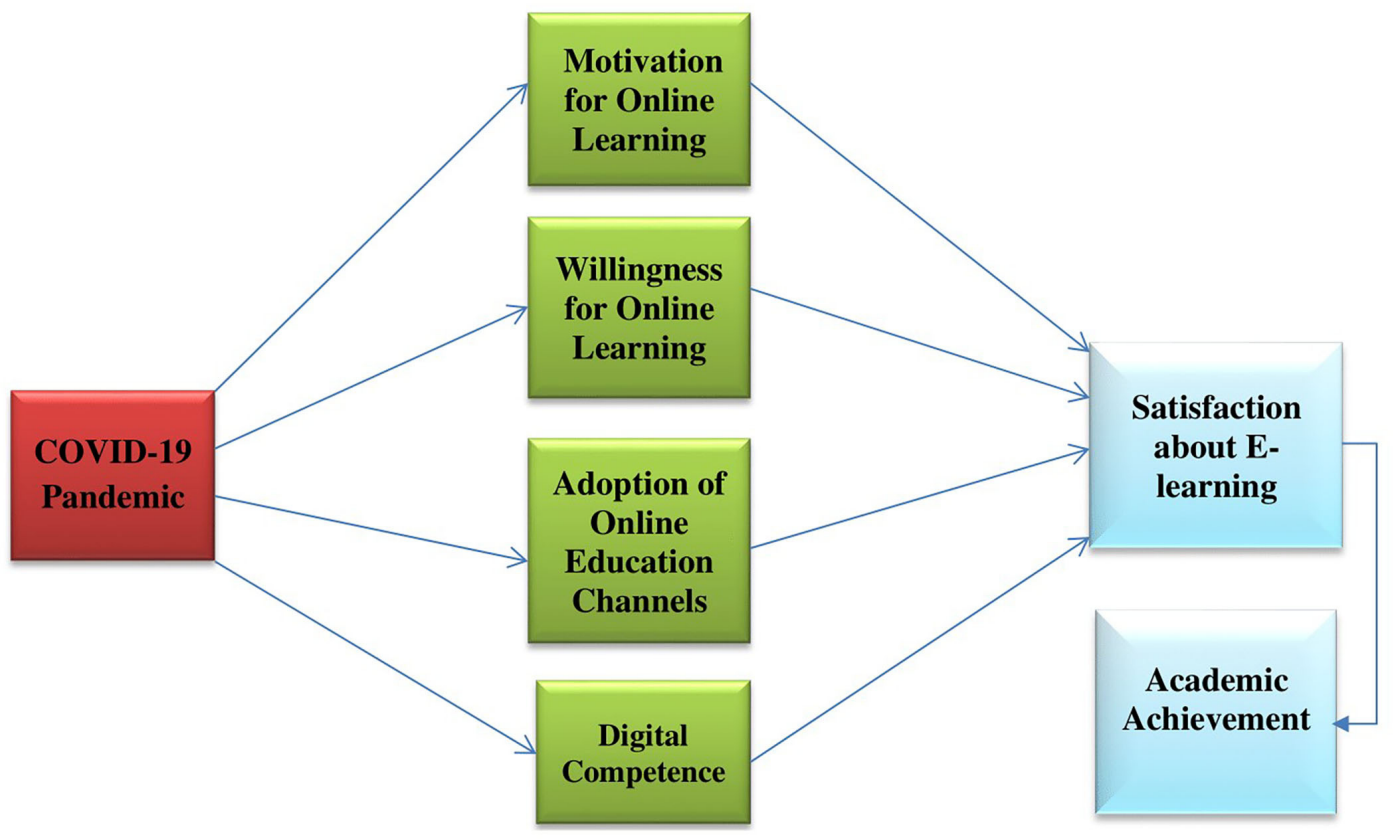
Study conceptual framework.

It is worth mentioning that students' satisfaction with e-learning is directly linked to their academic performance or achievement, and it may also be used to assess the effectiveness of online courses (Alqurashi, 2019). In a developing country like India, where device suitability and bandwidth availability are problems, e-learning planning, design, and effectiveness remain unknown (Muthuprasad et al., 2021). Understanding student satisfaction with e-learning and its relationship to motivation and willingness through adopting online channels and digital competence will primarily assist students in achieving better academic achievements. Perceived obstacles and COVID-19 knowledge directly affect students' intentions, but these impacts are further mediated by perceived utility and ease of use of e-learning technologies (Nikou and Maslov, 2021). The research is significant because it provides educators and policymakers with a fresh viewpoint on how to properly plan for the introduction of e-learning while keeping student satisfaction in mind. Hypothesized correlations between research variables are shown in Figure 1.

### Study hypotheses

**H1:** Motivation for online learning positively impacted the satisfaction about e-learning during the COVID-19 pandemic.

**H2:** Willingness for online learning positively impacted the satisfaction about e-learning during the COVID-19 pandemic.

**H3:** Adoption of online education channels positively impacted the satisfaction about e-learning during the COVID-19 pandemic.

**H4:** Digital competence positively impacted the satisfaction about e-learning during the COVID-19 pandemic.

**H5:** Academic achievement has a positive association with motivation for online learning.

**H6:** Academic achievement has a positive association with willingness for online learning.

**H7:** Academic achievement has a positive association with the adoption of online education channels.

**H8:** Academic achievement has a positive association with the digital competence.

## Research methods

### Study locale

The present investigation took place in Pakistan's Punjab province and was exploratory. The research was authorized by the Ethical Committee of Soochow University in Suzhou, Jiangsu before the final data was collected. The participants' involvement in the research was voluntary, and they gave their informed permission before the study began.

### Study design

This study used a questionnaire-based research approach to examine the association between Pakistani University students' e-learning satisfaction and academic achievement. The study took place in six cities in Punjab Province of Pakistan, 550 male and 650 female students with different age groups and educational backgrounds participated in this online survey and a well-designed questionnaire was used to obtain the main data. Five-point close-ended Likert scale questions were used in the survey.

### Participants

Between December 2021 and January 2022, the online survey of Pakistani University students was undertaken. Before the final survey, 50 respondents were pre-tested for response rate. After pre-testing, certain questions were changed to improve response rates. In total, 1,200 participant answers were evaluated after data quality assessment. Respondents have to meet the following criteria: (1) Pakistani University students who attended online courses during COVID-19 and (2) those who volunteered for this research. The convenient sampling technique was used to gather the primary data, and University students from six different cities of Punjab Province in Pakistan, namely, Lahore, Multan, Rawalpindi, Faisalabad, Bahawalpur, and Sialkot have fulfilled the inclusion criteria for this study.

### Operationalization of study variables

There are seven variables used in this study to investigate e-learning satisfaction and academic achievements of University students. The study confined one independent variable (COVID-19 Pandemic), four mediators (motivation for online learning, willingness for online learning, adaptation to online learning channels, and digital competence), and two dependent variables (satisfaction with e-learning and academic achievement).

### Demographic information

The demographic questions were about gender (male and female), age (20–25, 25–30, 30–35, 35+ years old), education (undergraduate, graduate, doctoral, or vocational degree), and year of attending the University (1–2, 3–4, +4 years).

### COVID-19 pandemic and online learning impact

Due to COVID-19, the students' online learning impact was calculated by adopting five items developed questions. Studying e-learning from students' and instructors' perspectives on using and developing e-learning systems at public universities while dealing with the COVID-19 epidemic has become more critical than ever before (Pokhrel and Chhetri, 2021; Maatuk et al., 2022). For this study, the courses were replaced with “online learning,” which impacted students' learning achievements. The participants used a five-point Likert scale ranging from 1 to 5.

### Students' satisfaction with e-learning

E-learning is a technology-driven distant learning tool. The studies sought to investigate undergraduate students' e-learning experience and identify variables impacting student and teacher satisfaction (Elshami et al., 2021; Giray, 2021). During the COVID-19 epidemic, when people were compelled to remain home, e-learning was the only way to learn. It is important to understand e-learners' perceptions and satisfaction with e-learning technologies.

## Data analysis and results

Structural equation modeling (SEM) of SmartPLS 3.0 was used to examine the measurement and structural models as it analyses intricate models that include both observable and latent components (Sharif et al., 2021). PLS-SEM may support SEM results with almost any degree of structural complexity, including higher-order structures that generally alleviate multicollinearity concerns (Ringle et al., 2015). The SmartPLS was used to evaluate the measurement and structural models and primary data can be analyzed using this tool (Hair et al., 2021). The SmartPLS research design is a reliable, scalable, and advanced method for developing a significant statistical model, and SmartPLS functions SEM's aid in achieving the intended goal (Abbas et al., 2019). SEM determines the model's discriminant, convergent, and average variance for each construct using factor loadings (Pahlevan Sharif and Mahdavian, 2015). Various connections between variables in the conceptual model may be studied using multivariate analytic methods.

### Descriptive statistics

The descriptive statistics in Table 1 displays that 54.17% of the study participants were female University students and 45.83% were male University students. The age of participants shows that the majority of the students were 20–25 (60.41%), 25–30 (18.33%), 30–35 (12.5%), and 35+ (8.75%) years old. Moreover, 70.83% of the participants were undergraduate students, 16.66% were graduates, 3.75% were PhD students, and 8.75% were with vocational degrees. Around 54.5% of students stated having four to five online classes per week and 21.33% of students reported three to four classes per week, and the rest of them reported one or two online classes weekly during a pandemic. Regarding their studying years in the University, 39.83% of them were in their first year and 41.66% were in their second year, and the rest of them were in their third and fourth years of studies.

**Table 1:** Demographic information of study participants (N-1200).

**Variables**	**Categories**	**Frequency/Percentage**
Gender	Male	550 (45.83%)
	Female	650 (54.17%)
Age	20–25	725 (60.41%)
	25–30	220 (18.33%)
	30–35	150 (12.5%)
	35+	105 (8.75%)
Education	Undergraduate	850 (70.83%)
	Graduate	200 (16.66%)
	Doctoral	45 (3.75%)
	Vocational	105 (8.75%)
Weekly online classes	1–2	88 (7.33%)
	2–3	103 (8.58%)
	3–4	256 (21.33%)
	4–5	654 (54.5%)
	5+	99 (8.25%)
Educational year in University	1st Year	478 (39.83%)
	2nd Year	500 (41.66%)
	3rd Year	130 (10.83%)
	4th Year	92 (7.66%)

### Multivariate analysis

#### Measurement model assessment

The statistical results of this study model are shown in Table 2. The reliability of the survey was measured using alpha values. According to Nunnally (1994) and She et al. (2021), the established value of alpha for measuring dependability is over 0.70, and each component is judged trustworthy according to the standard and Cronbach's alpha values ranging from 0.700 to 0.880. Composite reliability (CR) values for each construct were calculated ranging from 0.796 to 0.922. In this study, loading values routinely exceeded 0.60. The standard range of average variance extracted (AVE) is described as 0.50 (Anonymous, 2018). For discriminant validity, the square root of each construct's AVE should be greater than its connection with other constructs (Fornell and Larcker, 1981). This study's AVE values were more significant than the average range (from 0.500 to 0.797). To test multicollinearity, the variation influence factor (VIF) must be <1.000; 0.500 is acceptable. All formative constructions exhibited VIFs <0.800, indicating no multicollinearity.

**Table 2:** Results of the measurement model of research (N-1200).

**Constructs**	**Items**	**Loadings**	**VIF**	**Alpha**	**SCR**	**AVE**
COVID-19 pandemic	CP1	0.708	1.421	0.796	0.859	0.551
	CP2	0.753	1.926			
	CP3	0.710	1.483			
	CP4	0.800	1.976			
	CP5	0.734	2.495			
Motivation for online learning	MOL1	0.750	1.355	0.700	0.816	0.597
	MOL2	0.803	1.368			
	MOL3	0.763	1.213			
Willingness for online learning	WOL1	0.911	2.981	0.872	0.922	0.797
	WOL2	0.853	1.862			
	WOL3	0.913	2.924			
Adaptation of online education channels	AOEC1	0.854	1.973	0.734	0.850	0.655
	AOEC2	0.850	1.907			
	AOEC3	0.717	1.202			
Digital competence	DC1	0.894	2.495	0.787	0.876	0.706
	DC2	0.920	2.672			
	DC3	0.687	1.295			
Satisfaction about e-learning	SEL1	0.731	1.701	0.880	0.913	0.677
	SEL2	0.816	1.403			
	SEL3	0.888	3.107			
	SEL4	0.873	2.786			
	SEL5	0.796	2.214			
Academic achievement	AA1	0.794	1.116	0.712	0.796	0.500
	AA2	0.722	1.478			
	AA3	0.656	1.623			
	AA4	0.634	1.710			

#### Discriminant validity

**Figure 2:**
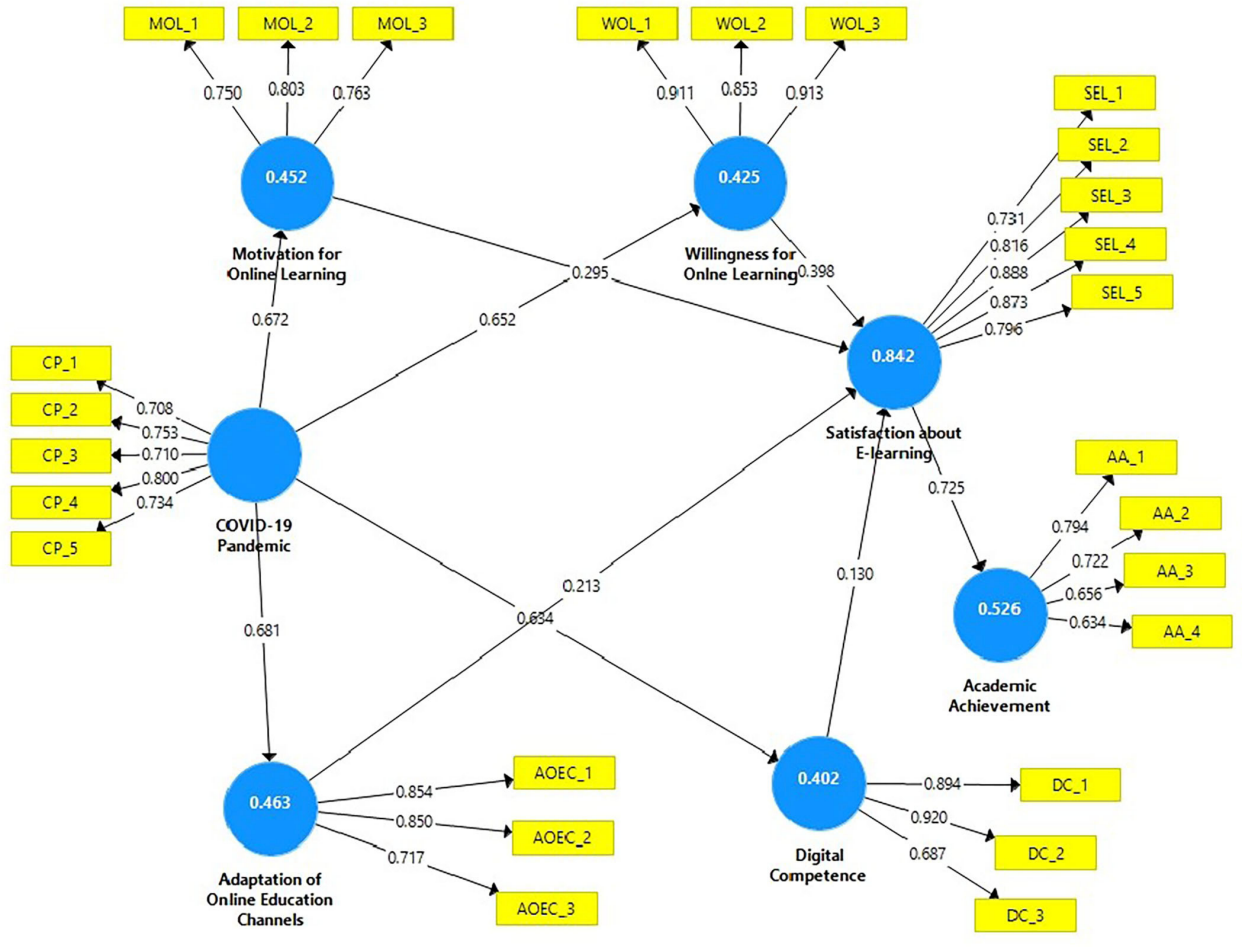
PLS-SEM.

It was shown that discriminant validity (DV) could be used to quantify concepts that had no connection to one another conceptually. Discriminant validation aims to show any evidence of discrimination based on the differences between all components (Campbell and Fiske, 1959). DV was used to evaluate and describe unrelated constructs. DV also gives the verification of all measures concerning component dissimilarity. When defining measure correspondence, DV entails evaluating non-statistically related components. DV may be calculated using a factor's AVE. The DV showed that the square root of each construct and AVE was greater than its connection with other constructs (Table 3; Figure 2).

**Table 3:** Discriminant validity assessment results (N-1200).

	**Academic achievement**	**Adaptation of online education channels**	**COVID-19 pandemic**	**Digital competence**	**Motivation for online learning**	**Satisfaction about e-learning**	**Willingness for online learning**
Academic achievement	0.704						
Adaptation of online education channels	0.545	0.809					
COVID-19 pandemic	0.533	0.681	0.742				
Digital competence	0.715	0.588	0.634	0.840			
Motivation for online learning	0.643	0.596	0.672	0.759	0.773		
Satisfaction about e-learning	0.725	0.738	0.713	0.792	0.810	0.823	
Willingness for online learning	0.675	0.684	0.652	0.784	0.726	0.861	0.893

### Structural equation model

It asserts that the structural model serves as the theoretical framework for using structural equations to assess the inner path model (Skrondal and Rabe-Hesketh, 2004; Chin, 2010). All assumptions were tested using (SEM) of SmartPLS 3.0. Model fitness was measured using standardized root-mean-square-residual (SRMR) which is a standardized-residuals index that evaluates model fitness, chi-square, and normed fit index (NFI) (Brown, 2006; Chen, 2007). The SRMR value compares observed covariance with predicted matrices. SRMR values of 0.08 or less may be used. The predicted SRMR value is 0.0510, which is adequate as a model fit. The NFI is 0.505, and the chi-square (2) value is 14086.307, as seen in Table 4.

**Table 4:** Model fit summary (N-1200).

	**Estimated model**
SRMR	0.0510
d_ULS	7.910
d_G	2.911
Chi-Square	14086.307
NFI	0.505

SRMR, Standardized-root-mean-square-residual; d_ULS, unweighted least squares discrepancy; d_G, geodesic discrepancy; X^2^ = chi-square; NFI, normed fit index.

**Figure 3:**
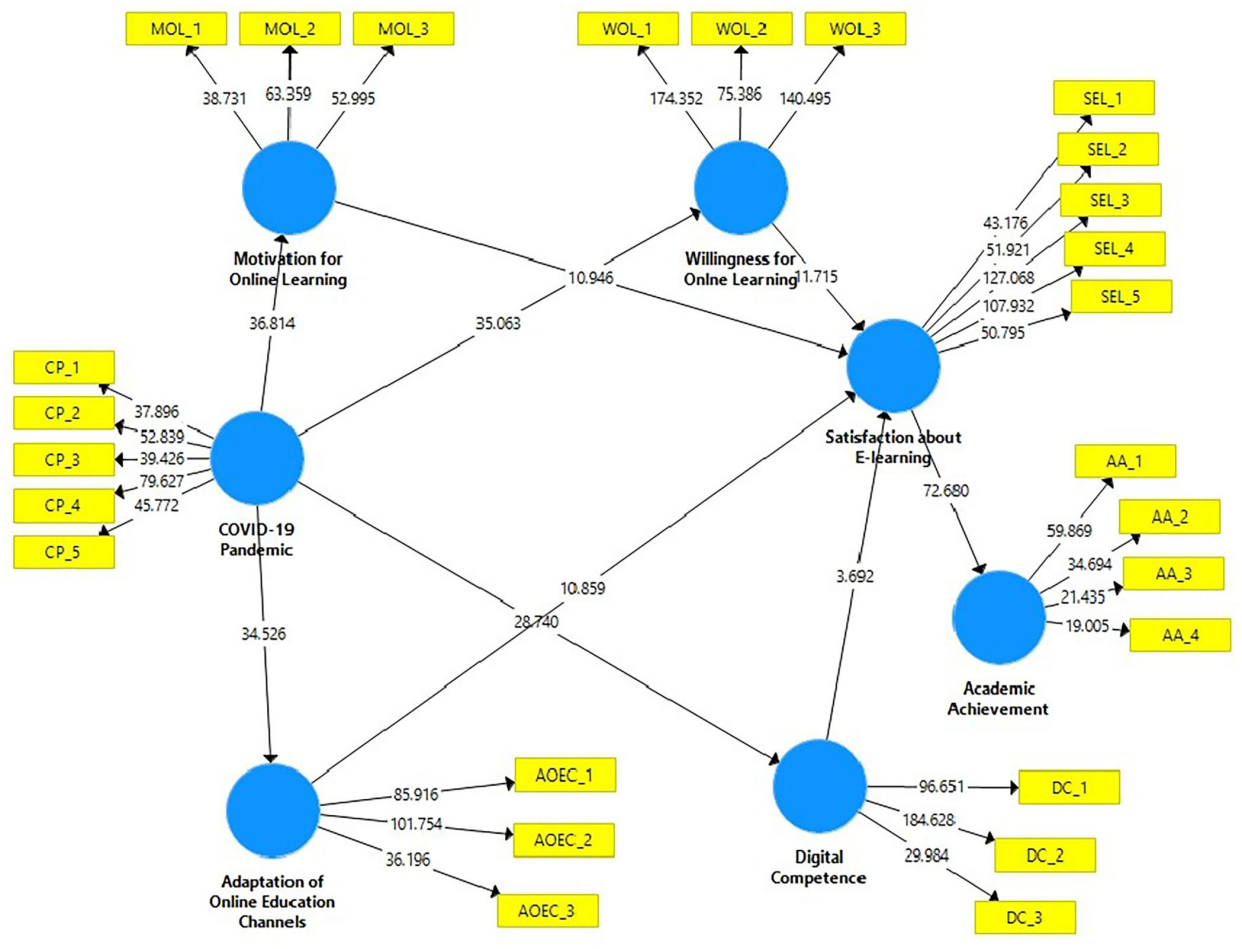
PLS-Bootstrapping, *T*-Values.

The standard beta was utilized to determine the significance of the hypotheses, and the beta value indicates how distinct variables may differ. The hypothesized research model was used to obtain the standardized beta (β) value for each connection (Table 5). The importance of endogenous latent variables will be judged crucial if beta (β) values are large and significant. The importance of each path's beta value was determined using T-statistics. The bootstrapping method was used to determine the significance of the beta (β) value and examine the relevance of assumed connections. Table 5 illustrates the recommended structural model connections and (β) statistics. SmartPLS-bootstrapping research variable *t*-values show the SmartPLS-bootstrapping *t*-values for the research variables (Figure 3).

**Table 5:** Final results of standard beta, t-statistics, and *p*-values (N-1200).

**Hypothesis's**	**Std. Beta (β)**	**T-Statistics**	***P*-Values**	**Decision**
Adaptation of online education channels -> Satisfaction about e-learning	0.213	11.637	0.0000	Confirmed
COVID-19 pandemic -> Adaptation of online education channels	0.681	35.083	0.0000	Confirmed
COVID-19 pandemic -> Digital competence	0.634	31.322	0.0000	Confirmed
COVID-19 pandemic -> Motivation for online learning	0.672	41.796	0.0000	Confirmed
COVID-19 pandemic -> Willingness for online learning	0.652	35.324	0.0000	Confirmed
Digital competence -> Satisfaction about e-learning	0.130	3.768	0.0000	Confirmed
Motivation for online learning -> Satisfaction about e-learning	0.295	11.228	0.0000	Confirmed
Satisfaction about e-learning -> Academic achievement	0.725	72.787	0.0000	Confirmed
Willingness for online learning -> Satisfaction about e-learning	0.398	11.606	0.0000	Confirmed

The result (HI: β = 0.213, t = 11.637, *p* = < 0.0000) reveals that the adaptation of online education channels have positive impact on satisfaction about e-learning. H2 results (β = 0.681, *t* = 35.083, *p* = < 0.0000) proved that COVID-19 pandemic has significant positive impact on the adaptation of online education channels. H3 results (β = 0.634, *t* = 31.322, *p* = < 0.0000) statistically reveals that COVID-19 pandemic has positive association with digital competence. H4 statistically documented that (β = 0.672, t = 41.796, *p* = < 0.0000) COVID-19 pandemic have positive effects on motivation for online learning. H5 supported that (β = 0.652, *t* = 35.325, *p* = < 0.0000) COVID-19 pandemic positively influences on students' willingness for online learning. H6 reveals that (β = 0.130, *t* = 3.768, *p* = < 0.0000) digital competence has positive association with satisfaction about e-learning. The result of H7 statistically documented that (β = 0.295, *t* = 11.228, *p* = < 0.0000) motivation for online learning has positive influence on satisfaction about e-learning. H8 results shows that (β = 0.725, *t* = 72.787, *p* = < 0.0000) satisfaction about e-learning has significant association with academic achievement and H9 reveals that (β = 0.398, *t* = 11.606, *p* = < 0.0000) willingness for online learning has positive association with satisfaction about e-learning.

## Discussion

This research investigates students' e-learning satisfaction and academic achievement to evaluate online influencing elements for learning among University students in Pakistan during the COVID-19 pandemic. The analysis of the SEM model shows that all planned hypotheses (H1: Adaptation of Online Education Channels -> Satisfaction about E-learning, β = 0.213, *t* = 11.637, *p* = < 0.0000; H2: COVID-19 Pandemic -> Adaptation of Online Education Channels, β = 0.681, *t* = 35.083, *p* = < 0.0000; H3: COVID-19 Pandemic -> Digital Competence, β = 0.634, *t* = 31.322, *p* = < 0.0000; H4: COVID-19 Pandemic -> Motivation for Online Learning, β = 0.672, *t* = 41.796, *p* = < 0.0000; H5: COVID-19 Pandemic -> Willingness for Online Learning, β = 0.652, *t* = 35.325, *p* = < 0.0000) are confirmed. Many students may suffer from psychological repercussions due to the sudden move from face-to-face classrooms and interactions with peers and professors to online learning (Lufungulo et al., 2021; Lim et al., 2022). During the COVID-19 pandemic, which had a significant impact on public health and education systems around the world, this study examines how public University students perceived online classes, measuring their research self-efficacy and course satisfaction before and after their procedures and results are consistent with previous studies (Randazzo et al., 2021; Sarkar et al., 2021). Since the COVID-19 pandemic, online teaching has been encouraged, all institutions have accepted it, and the COVID-19 pandemic has had an unparalleled influence on education globally. This gap has several dimensions, including access to gadgets and the internet and other external variables, such as parental support, teacher quality, and the learning environment (Coleman, 2021; Wu, 2021).

The findings revealed a link between student satisfaction with e-learning and academic accomplishment. According to the data, Pakistani students who used e-learning throughout the epidemic had greater learning satisfaction and academic achievement. The features of students' involvement in online learning remain understudied, and the factors impacting students' satisfaction and achievement in online courses during the COVID-19 pandemic era and the link between these variables have to be identified and established (Gopal et al., 2021; Salas-Pilco et al., 2022). All hypotheses (H6: Digital Competence -> Satisfaction about E-learning, β = 0.130, *t* = 3.768, *p* = < 0.0000; H7: Motivation for Online Learning -> Satisfaction about E-learning, β = 0.295, *t* = 11.228, *p* = < 0.0000; H8: Satisfaction about E-learning -> Academic Achievement, β = 0.725, *t* = 72.787, *p* = < 0.0000; H9: Willingness for Online Learning -> Satisfaction about E-learning, β = 0.398, *t* = 11.606, *p* = < 0.0000) statistically reveals the positive association in all. For future academics, several studies provide the directions and consequences for online education by identifying elements that impact online learning satisfaction at home as a result of the COVID-19 pandemic and getting information on difficulties in the teaching/learning process (Trisanti et al., 2021; Kornpitack and Sawmong, 2022). These studies provide a new understanding of learner interaction and its relationship to course content, teaching methods, and learning satisfaction in an Asian context. Students' satisfaction with online learning may be influenced by self-motivation and the usage of online tests as a form of evaluation (Basuony et al., 2020; Thach et al., 2021). By providing actual information on the components that encourage students' preparedness to continue online learning during the COVID-19 pandemic lockdown, new insights into the literature on students' desire to continue online learning were achieved (Mohd Satar et al., 2020; Ngah et al., 2022).

## Conclusion

With the increased use of e-learning during the pandemic period and the introduction of various technology applications and their impact on student satisfaction and academic achievement as online influencing factors, the entire domain of learning and education in Pakistan and around the world has changed as research indicated that e-learning has a significant relationship between student satisfaction and academic achievement. Pakistani students who utilized e-learning during the outbreak had higher learning satisfaction and academic achievement. The findings showed a correlation between e-learning satisfaction and academic achievement, with Pakistani students who used e-learning during the epidemic reporting better levels of academic achievement. The study is noteworthy because it offers educators and policymakers a new perspective on adequately preparing for the adoption of e-learning while ensuring student satisfaction.

### Study limitations and future scope

The study is not without limitations. The research sample consisted of University students from five renowned cities in the Punjab province of Pakistan, which did not reflect the whole population of Pakistani University students. Future studies may use longitudinal or experimental designs to give further evidence regarding observed correlations and their underlying processes. Future research should evaluate our concept in diverse circumstances, such as student e-learning satisfaction. A prospective study evaluating this approach may also include online influencing factors affecting students' satisfaction with e-learning.

## Data availability statement

The raw data supporting the conclusions of this article will be made available by the authors, without undue reservation.

## Ethics statement

The studies involving human participants were reviewed and approved by School of Education, Soochow University. The patients/participants provided their written informed consent to participate in this study.

## Author contributions

XQ is the principal investigator. MY collected data, wrote the article, and analyzed the data. XZ guided the psychological perspective and methodology. UN and RM designed the study model and hypothesis, contributed in discussion section, and proofreading and finalizing the manuscript. All authors contributed to the article and approved the submitted version.

## Funding

This study was supported by the Project Research on the Construction Quality of Research Groups in Universities in China approved by the National Office for Education Sciences Planning China (Project No: BIA200166).

## Conflict of interest

The authors declare that the research was conducted in the absence of any commercial or financial relationships that could be construed as a potential conflict of interest.

## Publisher's note

All claims expressed in this article are solely those of the authors and do not necessarily represent those of their affiliated organizations, or those of the publisher, the editors and the reviewers. Any product that may be evaluated in this article, or claim that may be made by its manufacturer, is not guaranteed or endorsed by the publisher.
